# TOPMed imputed genomics enhances genomic atlas of the human proteome in brain, cerebrospinal fluid, and plasma

**DOI:** 10.1038/s41597-024-03140-3

**Published:** 2024-04-16

**Authors:** Heng Yi, Qijun Yang, Charlie Repaci, Cheolmin Matthew Lee, Gyujin Heo, Jigyasha Timsina, Priyanka Gorijala, Chengran Yang, John Budde, Lihua Wang, Carlos Cruchaga, Yun Ju Sung

**Affiliations:** 1 Department of Psychiatry, Washington University School of Medicine, St. Louis, MO USA; 2 NeuroGenomics and Informatics Center, Washington University School of Medicine, St. Louis, MO USA; 3 Division of Biostatistics, Washington University School of Medicine, St. Louis, MO USA; 4 Institute for Informatics, Washington University School of Medicine, St. Louis, MO USA; 5 Hope Center for Neurologic Diseases, Washington University, St. Louis, MO USA

**Keywords:** Quantitative trait, Protein array analysis

## Abstract

Comprehensive expression quantitative trait loci studies have been instrumental for understanding tissue-specific gene regulation and pinpointing functional genes for disease-associated loci in a tissue-specific manner. Compared to gene expressions, proteins more directly affect various biological processes, often dysregulated in disease, and are important drug targets. We previously performed and identified tissue-specific protein quantitative trait loci in brain, cerebrospinal fluid, and plasma. We now enhance this work by analyzing more proteins (1,300 versus 1,079) and an almost twofold increase in high quality imputed genetic variants (8.4 million versus 4.4 million) by using TOPMed reference panel. We identified 38 genomic regions associated with 43 proteins in brain, 150 regions associated with 247 proteins in cerebrospinal fluid, and 95 regions associated with 145 proteins in plasma. Compared to our previous study, this study newly identified 12 loci in brain, 30 loci in cerebrospinal fluid, and 22 loci in plasma. Our improved genomic atlas uncovers the genetic control of protein regulation across multiple tissues. These resources are accessible through the Online Neurodegenerative Trait Integrative Multi-Omics Explorer for use by the scientific community.

## Introduction

Genome-wide association studies (GWAS) have successfully identified a large number of genetic variants associated with many human diseases^1^. Expression quantitative trait loci (eQTL) studies have been instrumental for understanding tissue-specific gene expression and regulation^2^. In particular, comprehensive and accessible catalogues provided by the Genotype-Tissue Expression (GTEx) project helped pinpointing functional genes for many disease-associated GWAS loci in a tissue-specific manner^3^. Compared to gene expressions, proteins more directly affect various biological processes, often dysregulated in disease, and are important drug targets. While several recent protein quantitative trait loci (pQTL) studies that identified genetic variants associated with inter-individual protein variability have uncovered intermediate molecular pathways for disease outcomes, they have been restricted to circulating plasma proteins^4,5^.

To address this knowledge gap, we previously obtained protein levels in neurologically relevant tissues—brain, cerebrospinal fluid (CSF), and plasma. By performing pQTL study, we subsequently identified tissue-specific pQTLs that were critical for understanding the biology of complex traits, particularly in neurological diseases^6^. Our previous pQTL study was evaluated at genetic variants imputed using the reference panel from the 1,000 Genomes Project, which consisted of sequence data from 2,504 individuals in human genome build 19 (HG19)^7^. Recently, the NHLBI Trans-Omics for Precision Medicine (TOPMed) project completed a deep sequencing of 53,831 individuals across diverse populations and provides a reference panel in human genome build 38 (HG38)^8^. This improved TOPMed reference panel provides an opportunity to impute more genetic variants with a better imputation quality for both rare and common variants.

In this study, we performed genotype imputation by using TOPMed reference panel and pursued multi-tissue pQTL study at these high-quality imputed genetic variants. By analyzing more proteins and an almost twofold increase in high-quality imputed genetic variants, we identified 38, 150 and 95 genomic regions associated with 43 proteins in brain, 247 proteins in CSF, and 145 proteins in plasma, respectively. These pQTL findings are assessable through the Online Neurodegenerative Trait Integrative Multi-Omics Explorer (ONTIME; ontime.wustl.edu/) for the scientific community.

## Methods

### Data sources

All the data sets used in this study are openly available from the National Institute on Aging Genetics of Alzheimer’s Disease Data Storage Site (NIAGADS). In particular, the Knight-ADRC repository (https://www.niagads.org/knight-adrc-collection) were created for the Knight Alzheimer’s Disease Research Center (Knight ADRC)^9^ Memory and Aging Project at Washington University School of Medicine. NIAGADS is a secure storage and sharing site for NIH-funded genetic studies and is appropriate to host our sensitive data. The NIAGADS Data Sharing Service (DSS) utilized cloud technology and was complaint with both Health Insurance Portability and Accountability Act (HIPAA) and the Federal Information Security Management Act of 2002 (FISMA). This study utilized genomics data (accession number NG00127.v1), which is available in https://dss.niagads.org/datasets/ng00127, and proteomics data (accession number NG00102.v1), which is available in https://www.niagads.org/datasets/ng00102. Both data sets can be obtained through NIAGADS. The Institutional Review Board (IRB) of Washington University School of Medicine in St. Louis approved the study with IRB number 201109148, and research was performed in accordance with the approved protocols.

### Genomic data, QC and imputation

Knight ADRC samples had been genotyped on multiple Illumina platforms (spanning 10 years). As a part of quality control (QC), we considered SNPs and individuals with genotyping rate of at least 98% per SNP or per individual and Hardy-Weinberg equilibrium (HWE) test (with P ≥ 1 × 10^−6^). We checked the consistency between sex of individuals and that estimated by genotype data and excluded those individuals with inconsistent sex information. Specifically, this sex check was performed using PLINK with the “check-sex” option, which provides SNPSEX, genetically determined sex based on the heterozygosity rates of X chromosome data. If the reported sex was inconsistent with this SNPSEX, the sample was removed.

Before imputation, genome coordinates from hg19 were lifted over to hg38 using liftOver package in R^10,11^. We subsequently imputed using TOPMED (Version R2 on GRC38)^12^ with Eagle haplotype phasing (version 2.4). Only autosomal variants were imputed. Imputed variants were removed if an imputation quality score was less than 0.3, the call rate was less than 98%, or not in HWE. In addition, performed a relatedness check using identity by descent (IBD) and included only those unrelated individuals. We uploaded these imputed data (accession number NG00127.v1) to the NIAGADS (https://dss.niagads.org/datasets/ng00127/). A list of all uploaded files is shown in Supplementary Table 1.

### Proteomic data

Proteomic data (accession number NG00102.v1) contained data in parietal lobes, CSF and plasma from the Knight ADRC samples. These were obtained through multiplexed, aptamer-based SOMAscan platform using 1,305 modified aptamers^13^. Laboratory staff obtaining proteomic assays were blinded to the genotypes of participants. SomaLogic performed QC at the sample and aptamer level including hybridization control normalization, median signal normalization and inter-plate calibration using control aptamers (positive and negative controls) and calibrator samples. For each sample, hybridization controls on each plate were used to correct for systematic variability in hybridization. To correct for within-run technical variability, the median signal over all aptamers was assigned to different dilution sets within each tissue. The resulting hybridization scale factors and median scale factors were used to normalize data across samples within a run. The calibrator samples were used to correct for between-run variability.

To restrict our analysis to unrelated individuals with European ancestry, we performed principal component analysis (PCA) after merging the high-quality genomic data of Knight ADRC participants and the sequencing data from the 1000 Genomes Project (1KG)^14^, downloaded from http://ftp.1000genomes.ebi.ac.uk/vol1/ftp/data_collections/1000_genomes_project/release/20181203_biallelic_SNV/ (11/29/2021 release). PLINK^15^ was used to compute the first ten PCs. Based on the scatter plot between first two PCs, we selected Knight ADRC samples that were genetically similar to European individuals from the 1KG data (Supplementary Figure 1). In addition, these computed PCs were subsequently used as covariates in the GWAS analysis to correct for any possible bias due to population stratification. We considered proteomic data of 1,300 proteins for 378 individuals in brain, 869 proteins for 816 individuals in CSF, and 953 proteins for 529 individuals in plasma in this study. We uploaded these data (accession number NG00102.v1) to the NIAGADS (https://dss.niagads.org/datasets/ng00102/). The demographic data for the samples, including age, sex, and ten PCs, were uploaded with same access number. A list of all uploaded files is shown in Supplementary Table 2.

### Multi-tissue pQTL mapping

By integrating proteomic and genomic data, we performed GWAS for protein levels for each autosomal variant using glm option in PLINK2^15^ version v2.00a2.3LM, including age, sex, 10 genetic principal components (PC), and genotype array information as covariates. A total of 1,271 individuals with 160,506,717 imputed and directly genotyped variants were used for this study. Protein levels were log10 transformed to approximate the normal distribution. The distributions of 1300 proteins in brain, 869 proteins in CSF, and 953 proteins in plasma are presented in Supplementary Figures 2–4, respectively. All proteins used in analysis are summarized in Supplementary Table 3.

Significant association was classified into *cis*- and *trans*-pQTLs based on the following criteria. If the variant was within 1 Mb upstream or downstream of the transcription start site (TSS) with a P < 5 × 10^−8^, it was classified as local-acting *cis*-pQTL. If the variant was outside the *cis* region ( ± 1 Mb of TSS) at a study-wide significance (P < 5 × 10^−8^/number of proteomic PCs), the association was classified as *trans*-pQTL. The minimum number of PCs needed to explain 95% of the variance in proteomic data was calculated and used. They corresponded to 105, 228, and 240, for brain, CSF, and plasma, respectively, resulting in the P thresholds as 4.67 × 10^−10^, 2.19 × 10^−10^, and 2.08 × 10^−10^; Table 1). The coding genes were annotated by UniProt identifiers^16^ and TSS information for each gene was annotated by R package ‘biomaRt’^17^ with GRCh38.p13.

**Table 1:** Characteristics of genomic and proteomic data used in the current study and those used in the previous study (Yang *et al*., 2021).

Study*	Tissue	# Subjects	# Proteins	# Indep. proteins**	*trans* pQTL threshold***	MAF threshold	Tested genetic variants
This study	Brain	378	1,300	105	4.67 × 10^−10^	0.01	8.30 million
	CSF	816	869	228	2.19 × 10^−10^	0.005	9.50 million
	Plasma	529	953	240	2.08 × 10^−10^	0.01	8.48 million
Previous study	Brain	343	1,079	75	6.67 × 10^−10^	0.02	3.70 million
	CSF	817	713	169	2.96 × 10^110^	0.02	4.37 million
	Plasma	528	931	230	2.17 × 10^−10^	0.02	4.40 million

*This study performed GWAS at the genetic variants imputed based on the TOPMed reference panel, whereas the previous study (Yang *et al*., 2021) performed GWAS at the variants imputed based on the 1000 Genomes Project.

**The number of independent proteins corresponded to the number of principal components (PCs) that explains 95% of variance in proteomics data.

**The threshold for *trans* pQTL corresponded to 5 × 10^−8^ divided by the number of independent proteins. The threshold for *cis* pQTL was genome-wide (5 × 10^−8^)

All significant pQTLs were annotated using ANNOVAR^18^ version 2018-04-16 with the geneanno function in gene-based annotation mode. Genomic features and variants affecting the nearest genes were used for downstream analyses. To transfer variant position ID to reference SNP ID (rsID) from dbSNP, VarNote^19^ was utilized. The Target name, UniProt ID, EntrezGene ID, and Organism information were from the annotation file provided by SomaLogic.

### Disentangling independent signals in a locus

To identify independent signals within each pQTL, we performed stepwise conditional analysis. For each round, significant variants were selected at the significance threshold P < 5 × 10^−8^. Before conditioning (round 0), each index variant (i.e, a variant with the smallest P in the region) was selected. Then, variants in 1 Mb upstream or downstream of the index signal were clumped using clump function in PLINK1.9^15^ version v1.90b6.4. For the next rounds, variants that passed the significance threshold were included in the analysis and the index signal in the region was included as an additional covariate. The rounds repeated until there was no variant passing the significance threshold. When the analysis was done, the results were visualized using LocusZoom version 1.3^20^.

### Pleiotropic loci

Any significant region associated with more than one protein was identified as a pleiotropic region. In order to minimize any influence from LD, independent LD regions in hg38 (Berisa-Pickrell regions^21^, lifted over) were defined based on European LD scores from the 1000 Genomes Project Phase 3 data for the HapMap3 SNPs. All significant variants were assigned into a single region per LD (EUR)-defined loci for each tissue. The 2-Dimensional Manhattan plots were generated using functions from the R package ggplot2. Circos plots were generated using functions from the R package circlize^22^.

### Mendelian randomization and colocalization

We performed a two-sample Mendelian randomization (MR) analysis to estimate the causal effect of proteins on Alzheimer’s disease (AD) risk by utilizing genetic variants as instrumental variables. The latest AD GWAS summary statistics were downloaded from the NHGRI-EBI GWAS Catalog^23^ for study GCST90027158^24^. MR analysis was conducted with functions from TwoSampleMR^25^ package in R. To reduce the potential bias in our MR analysis, we removed pleiotropic regions. Also, we selected independent variants as instrumental variables after clumping (clump_r2 = 0.001, clump_kb = 500). Additionally, we performed a harmonization process with harmonise_data function using default options to combine datasets from different sources. The Wald ratio was used to estimate the causal effects. To determine significant pQTLs with a causal effect on the outcome, we corrected for false discovery rate (FDR) with a threshold of p-value < 0.05. Finally, we created regional plots using locuszoom^20^ to visualize the significant pQTLs and those for AD GWAS.

To investigate whether there is a shared causal variant between AD GWAS and pQTLs at a specific locus and to provide additional evidence for MR results, we conducted a Bayesian co-localization analysis. For this analysis, we utilized the coloc.abf function in the R package coloc^26,27^. Initially, we selected regions where the distance between the AD GWAS index signals and pQTL index signals was less than 2 Mb. We chose all the variants within a 1 Mb region ( ± 500Kb) from the index signal for the co-localization analysis. We used posterior probability for hypothesis 4 (PP.H4) indicating the presence of a single causal variant affecting the two traits. If PP.H4 was greater than 0.8, we concluded that the same functional variant affects both AD GWAS and pQTL at that locus.

### Comparison with findings from the 1000 Genomes imputed data

We compared this study with our previous study that were based on the 1000 Genomes imputed data^7^. NCBI Genome remapping was used to covert genome coordinates between HG38 and HG19. Our main focus was to compare the number and detailed information of significant variants, regions, and independent pQTLs between the two studies. We defined loci replication as the inclusion of top signals in the HG38 region within the 2 Mb window of the HG19 region.

### Web browser for navigating GWAS and PheWAS results

The Online Neurodegenerative Trait Integrative Multi-Omics Explorer (ONTIME) (available at https://ontime.wustl.edu) is a web browser that we developed using PheWeb version 1.3.16^28^, an open-source tool for visualizing and sharing GWAS and PheWAS results. We have now extended this browser to include results from this pQTL study. One of the key features of ONTIME is its interactive plot, which displays pQTL data and allows users to explore the data in detail. ONTIME provides intuitive visual summaries at three levels of detail: genome-wide summaries with traits, regional view, and phenome-wide associations. For GWAS genome-wide summary results, we utilized Manhattan and QQ plots. For a regional view, we used LocusZoom to display the LD among the variants in the region near the gene. Finally, phenome-wide summaries were utilized to highlight the association and P at the genetic variant across all proteins. All figures generated by ONTIME can be downloaded by users for further analysis.

## Results

### Multi-tissue pQTL mapping with TOPMed imputed genomics

Proteomic data of 1,300 proteins for 378 individuals in brain, 869 proteins for 816 individuals in CSF, and 953 proteins for 529 individuals in plasma were used for this study. Based on genetic principal components (PC) of samples, we restricted our analysis to unrelated individuals with European ancestry. The TOPMed imputed data provided about 9.5 million variants with minor allele frequency (MAF) over 1% (or 0.5% in CSF which contains more individuals) for 770 Knight ADRC samples with CSF proteomics data (Table 1). Our previous study using genomic data imputed with the 1000 Genomes Project reference panel provided 4.4 million variants with MAF over 0.02 for the same 770 samples. Because of an improved imputation panel, this study examined association for all variants with MAF over 1% (or 0.5%). The number of tested variants was twice larger across all three tissues than the previous data (8.3 million versus 3.7 million in brain; 9.5 million versus 4.37 million in CSF; and 8.48 million versus 4.4 million in plasma; Table 1).

**Fig. 1:**
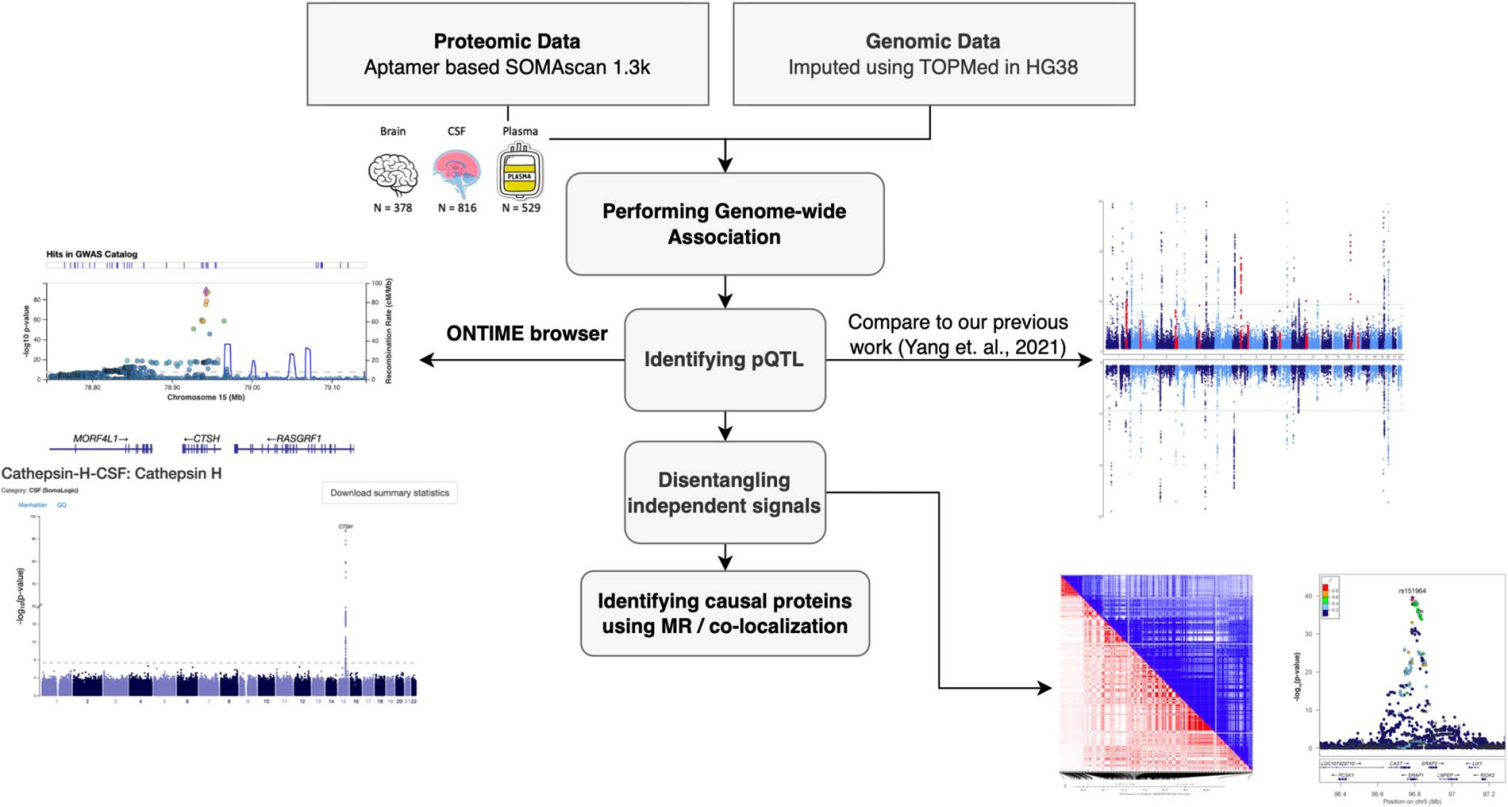
Study overview. Proteomic data in three tissues and genomic data imputed with TOPMED were obtained and integrated to perform GWAS. Protein QTL (pQTL) were identified and further characterized with conditional analysis and pleiotropic regions. These results were compared with findings from our previous study and included in the ONTIME web browser.

**Fig. 2:**
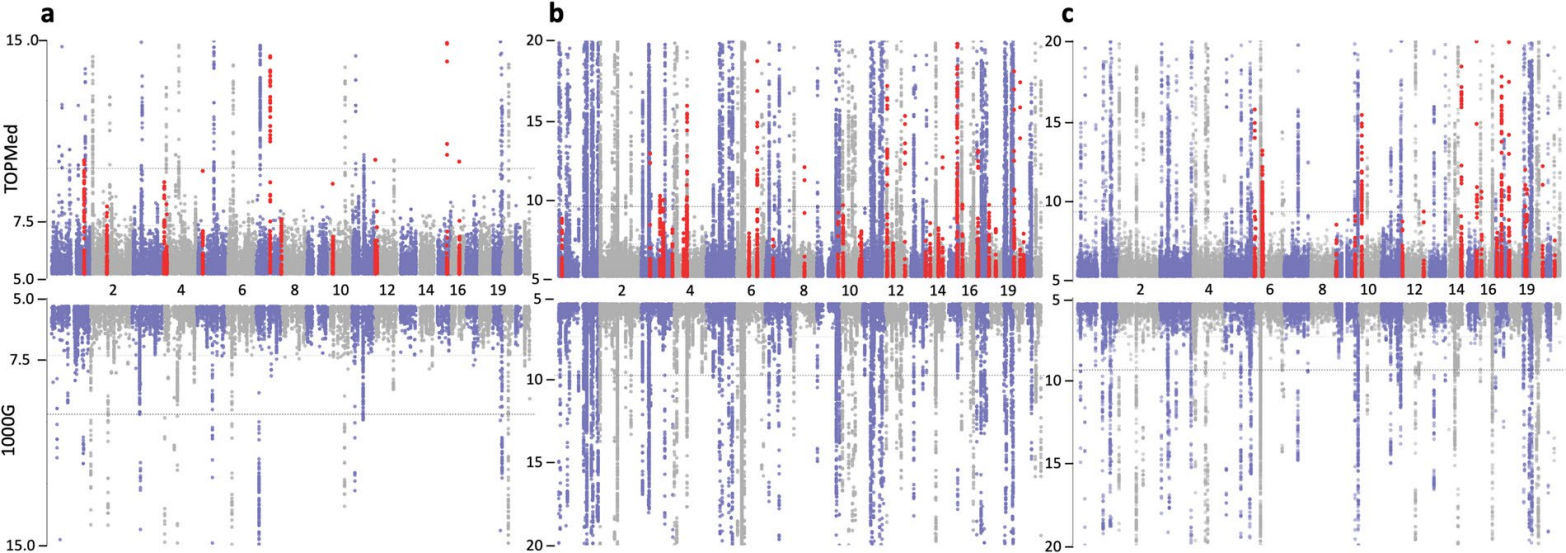
Improvement with TOPMed imputed data. Miami plots comparing pQTL findings from this study (upper) with findings from our previous study (lower) in brain (panel a), CSF (panel b), and plasma (panel **c**). Newly identified hg38 findings were shown in red. The y-axis was restricted to P > 1.0 × 10^−15^ in brain and P > 1.0 × 10^−20^ in CSF and plasma.

We performed GWAS for 3,122 proteins (1,300 in brain^29–32^; 869 in CSF^33–35^; 953 in plasma^36–39^), where each GWAS result provided an association between a protein and each of about 9 million tested genetic variants (Fig. 1). This study identified substantially more pQTL than our previous work^6^. In brain analysis, we found 3,131 significant associations for 43 proteins in 38 genomic regions (Uploaded Tables 1, 2), where each region is defined as 1 Mb upstream or downstream of the index signal. In CSF, there were 38,774 associations for 247 proteins in 150 genomic regions (Uploaded Table 2). In plasma, there were 13,344 associations for 145 proteins in 95 genomic regions (Uploaded Table 3). We generated the Miami plots (Fig. 2) that compare the findings from this study with the previously reported results. Among the 38 pQTL in brain, 26 were reported previously^6^ and 12 loci were newly identified (shown in red in Fig. 2). We found 30 newly identified pQTL in CSF and 22 in plasma (Table 2). The number of significant pQTL was affected by the sample sizes, as a larger sample at more variants (for example in CSF) provides more statistical power for identifying association.

**Table 2:** The number of significant associations, pQTL and independent signals.

Tissue	Study*	Significant associations	Genomic regions with pQTL**	Independent signals***
Brain	This study	3,131	38	40
	Previous study	2,484	26	32
	Additional findings	1,038	12	12
CSF	This study	38,774	150	219
	Previous study	25,993	127	174
	Additional findings	15,076	30	31
Plasma	This study	13,344	95	124
	Previous study	9,710	73	90
	Additional findings	5,515	22	31

*This study performed GWAS at the genetic variants imputed based on the TOPMed reference panel, whereas the previous study (Yang *et al*., 2021) performed GWAS at the variants imputed based on the 1000 Genomes Project.

**Genomic regions showing pQTL (*cis* or *trans*) associated with at least one of proteins.

***For each genomic region, independent signals were obtained from the conditional analysis.

### Disentangling independent signals within each locus

**Fig. 3:**
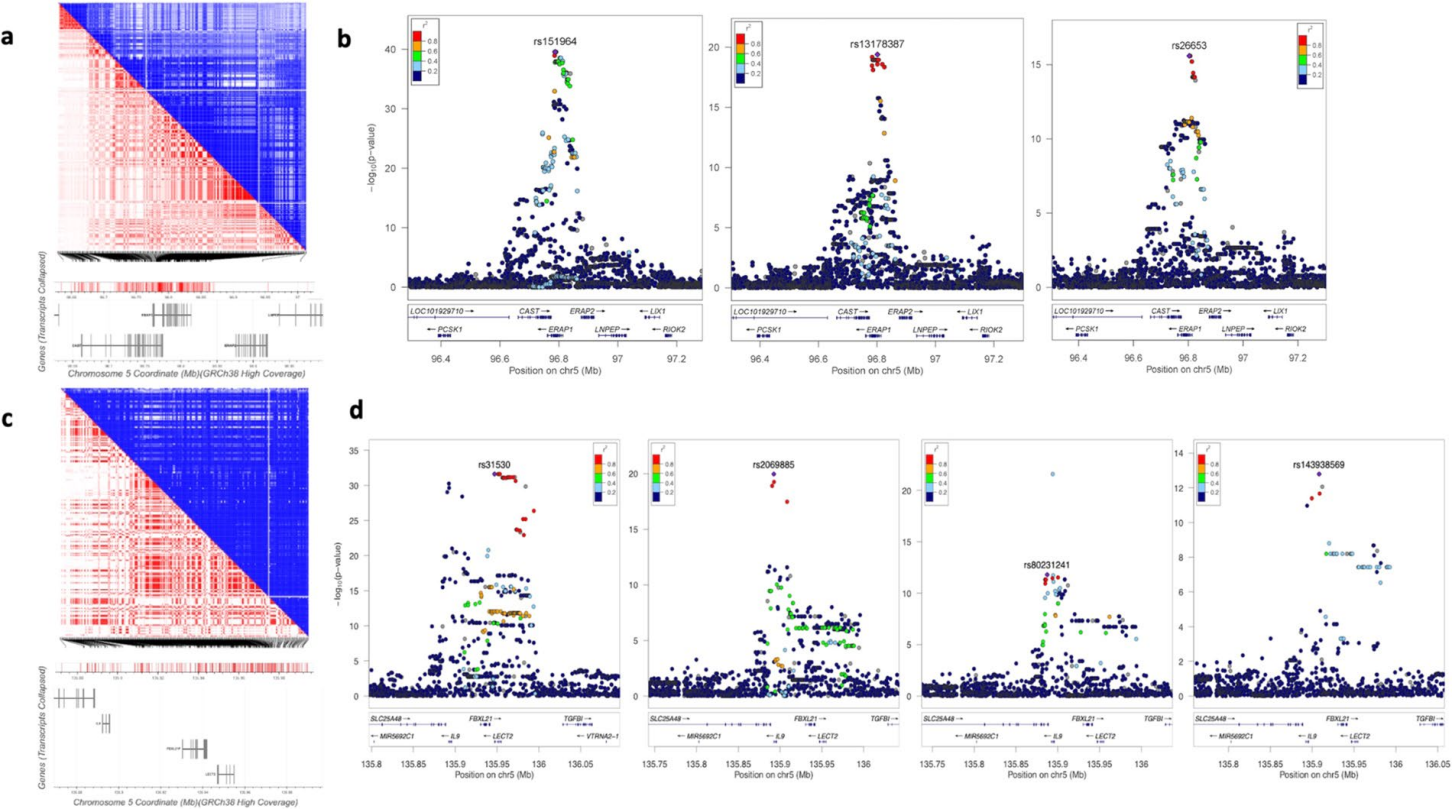
Complexity of pQTL in *ERAP1* and *LECT2* regions. Brain *cis* pQTL for ARTS1 in *ERAP1* contains 279 variants at P < 5 × 10^−8^ belonging to multiple LD blocks (**a**), resulting in three independent signals (local plots in **b**). CSF *cis* pQTL for Interleukin-9 in *LECT2* contains 257 genome-wide significant variants (LD in **c**), resulting in four independent signals (local plots in **d**).

Each pQTL may contain multiple independent variants associated with protein levels. To identify such independent signals within each pQTL, we performed a conditional association analysis for each locus by including the sentinel (top index) variant as an additional covariate. When multiple association signals were present, we continued this iteratively until no associations remained. For example, *cis* pQTL for ARTS1 in brain contained 279 genetic variants reaching genome-wide significance (all with P < 5 × 10^−8^; Uploaded Table 1; Fig. 3). The sentinel variant was observed at the common variant (rs151964, MAF = 0.36, β = 0.14, P = 2.69 × 10^−40^) located in an intron of *ERAP1*. There was a missense variant (rs30187) in LD (r^2^ = 1). After conditional analysis, we identified a secondary signal at another common variant (rs13178387, MAF = 0.20) also in intron of *ERAP1* (β = −0.15, P = 3.50 × 10^−32^ before conditioning; β = −0.10, P = 4.01 × 10^−20^ after conditioning). Another missense variant (rs2287987) was in LD (r^2^ = 0.8). Additional conditional analysis identified a third signal at rs26653, a missense variant in *ERAP1* (β = 0.14, P = 2.90 × 10^−39^ before conditioning; β = 0.08, P = 2.55 × 10^−16^ after conditioning; Fig. 3). All the remaining 37 pQTL in brain contained one independent signal.

In CSF, 47 pQTL had more than one independent signals. There were 29 pQTL with two signals, 12 pQTL with three independent signals, 6 pQTL with four independent signals. For example, *cis* pQTL for Interleukin-9 in CSF contained 257 genome-wide significant variants (Uploaded Table 2; Fig. 3). This locus had four independent signals. The primary signal was observed at the common variant (rs31530, MAF = 0.37, β = −0.08, P = 2.42 × 10^−32^) located at the UTR of *LECT2*. There was a missense variant (rs31517) in LD (r^2^ = 0.94). The secondary signal was observed at a missense variant (rs2069885, MAF = 0.12, β = −0.12, P = 5.42 × 10^−31^) in *IL9*. The third signal was observed at the intronic variant (rs80231241, MAF = 0.05, β = 0.13, P = 1.95 × 10^−17^) in *SLC25A48*. Finally, the fourth independent signal was observed at low frequency variant (rs143938569, MAF = 0.02, β = 0.18, P = 4.64 × 10^−12^), located between *IL9* and *FBXL21P*. The remaining 103 pQTL belonged to one single LD block. In plasma, there were 72 pQTL with one independent signal, 17 pQTL with two signals, and 6 pQTL with three independent signals.

### Pleiotropic loci

**Fig. 4:**
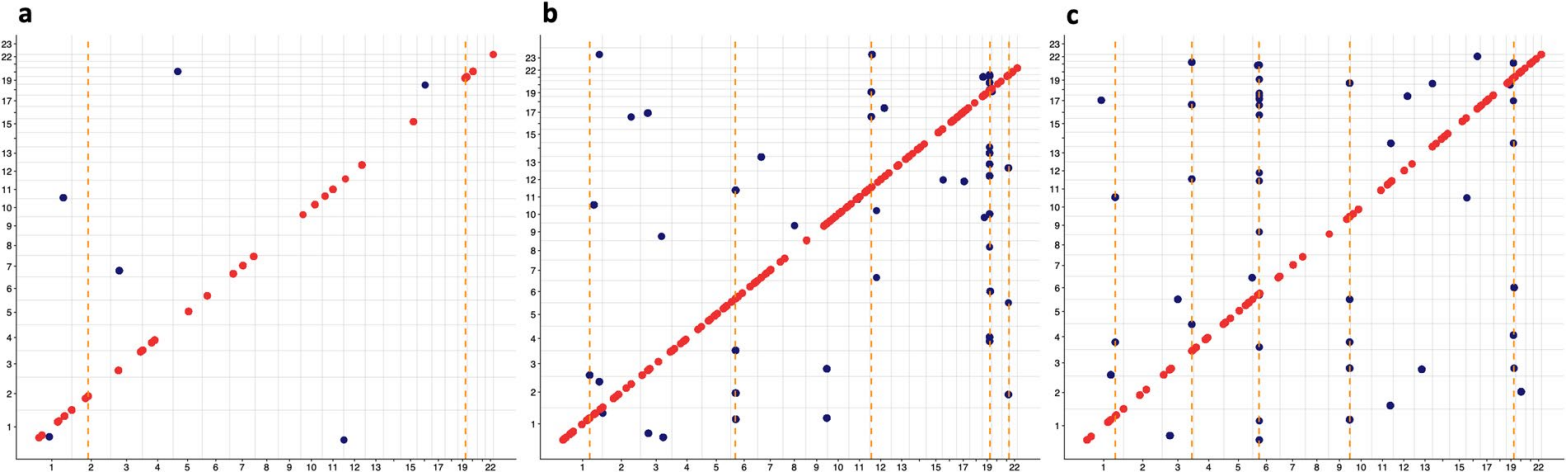
Global two-dimensional view of pQTL mapping. We identified both *cis* pQTL (red points) and *trans* pQTL (blue points) in brain (**a**), CSF (**b**), and plasma (**c**). The x-axis is the position of genetic variants regulating the protein levels. The y-axis is the location of transcription start site (TSS) of the gene encoding the protein for the pQTL signal.

To separate local-acting *cis* pQTL from *trans* pQTL, we generated a two-dimensional bird’s-eye view of association identified in this study (Fig. 4). Of the 150 associated regions in CSF, 130 (86.7%) had *cis* pQTL only, 16 (10.7%) *trans* only, and 4 (2.6%) both *cis* and *trans*. In plasma, 78 (82%) had *cis* only, 14 (15%) *trans* only and 3 (3%) both. The genomic regions in brain included 32 *cis* pQTL and 6 *trans* pQTL.

**Fig. 5:**
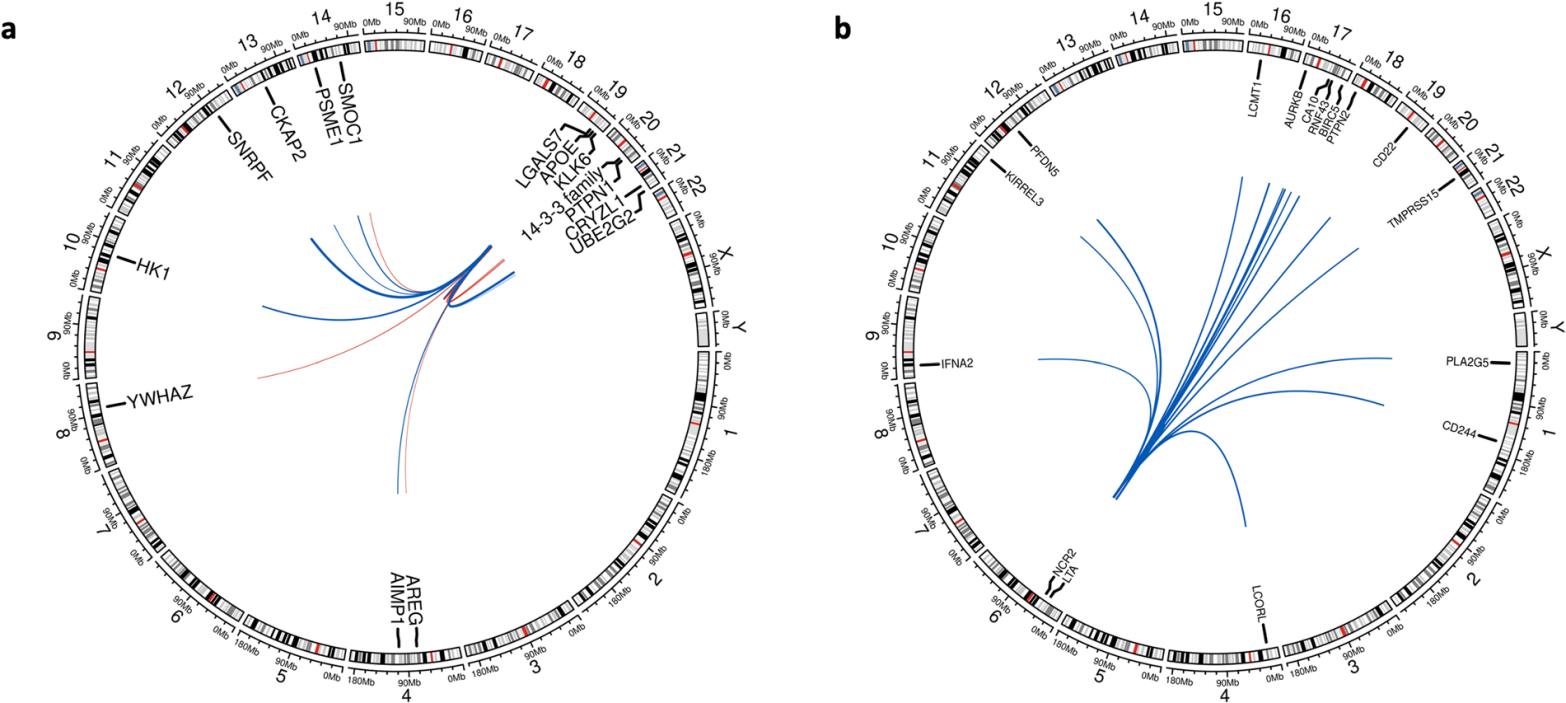
Circos plots of pleiotropic regions. The *APOE* locus on chromosome 19 was associated with 15 proteins in CSF (**a**), and the major histocompatibility complex (MHC) locus on chromosome 6 was associated with 16 proteins in plasma (**b**). Lines link the genomic location of the variant with genes encoding the associated proteins. Line thickness is proportional to effect size of association (red, positive; blue, negative).

**Fig. 6:**
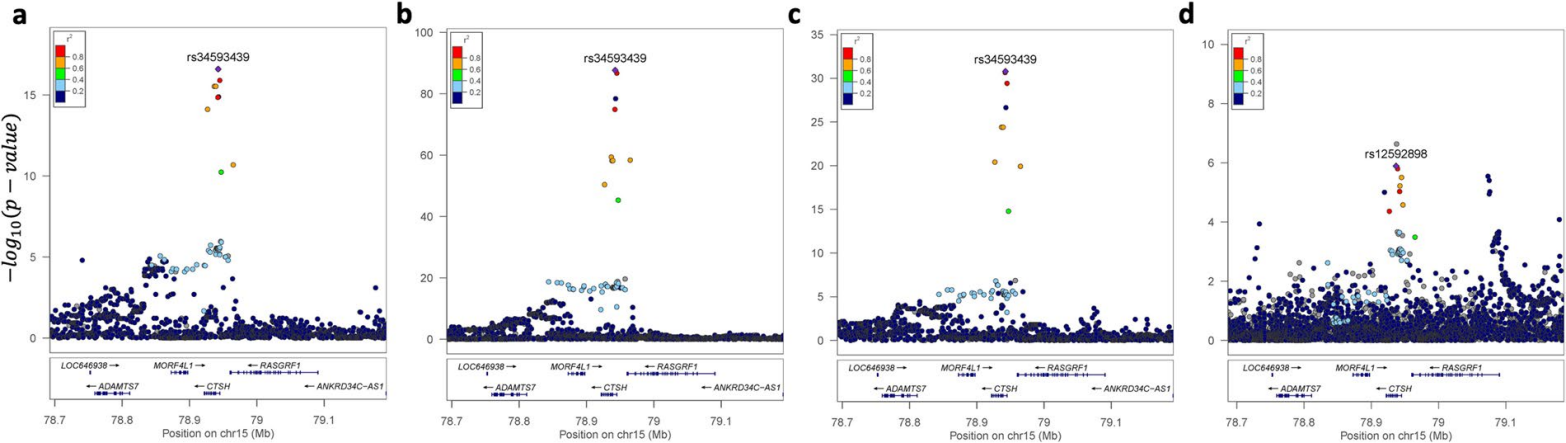
Colocalization of Cathepsin H with AD risk across three tissues. Cathepsin H showed the evidence of being causal (indicating that higher Cathepsin H levels significantly increase AD risk) and colocalized (PP.H4 > 0.94) with AD risk at *CTSH*. Local association plots of Cathepsin H are shown for brain (**a**), CSF (**b**), and plasma (**c**) along with the local plot of AD risk (**d**).

While most regions were associated with a single protein, we found several pleiotropic loci, genetic regions that were associated with multiple proteins. In CSF, there were 49 pleiotropic loci (Supplementary Table 4), where 6 loci were associations with more than five proteins. In particular, the *APOE* locus on chromosome 19 was associated with 15 proteins (Fig. 5). In brain, there were 33 pleiotropic loci, including 3 loci associated with more than 5 proteins. In plasma, there were 21 pleiotropic loci, where 3 loci were associated with more than 5 proteins. This included the major histocompatibility complex (MHC) locus on chromosome 6 that were associated with 16 proteins (Fig. 6). In brain, there were 4 pleotropic loci, including the *SIGLEC* gene cluster on chromosome 19.

### Mendelian randomization and colocalization

As a proof of concept, we investigated whether any of proteins with pQTL would be causal for Alzheimer’s disease (AD). We found that 5 proteins in brain, 10 proteins in CSF, and 24 proteins in plasma had evidence of being causal for AD risk (Supplementary Table 5). This is more than what we previously found (7 proteins in brain, 3 in CSF, and 13 in plasma)^6^. In addition to more variants tested, this study considered 75 AD loci from Bellinguez *et al*.^24^, whereas the previous study considered the 21 AD loci from Kunkle *et al*.^40^, the most comprehensive AD GWAS at the time of publication. For each of these potentially causal proteins, we further examined a presence of one single functional variant affecting both protein levels and AD risk with Bayesian colocalization method, coloc R package^27^. We found colocalization evidence (with posterior probability PP.H4 > 0.8) for two proteins in brain (Cathepsin H and Siglec-9). There was such colocalization evidence also for five proteins in CSF and one protein in plasma (Supplementary Table 6).

In all three tissues, Cathepsin H showed the evidence of being causal and colocalized with AD risk (Fig. 6). The minor allele (A) of the top sentinel variant rs34593439, located in intron of *CTSH*, was associated with lower Cathepsin H levels, consistently in all three tissues (β = −0.18, P = 2.47 × 10^−17^ in brain; β = −0.26, P = 2.03 × 10^−88^ in CSF; β = −0.23, P = 1.59 × 10^−31^ in plasma). The latest AD GWAS newly identified *CTSH* locus associated with AD risk^24^. The minor allele (A) of the index variant rs12592898 was associated with lower AD risk (OR = 0.94, P = 4.2 × 10^−9^). These two index variants were in moderate LD (r^2^ = 0.78). Our MR results showed a causality of Cathepsin H levels for AD risk with positive relationship in all three tissues (β = 0.34, FDR = 1.10 × 10^−4^ in brain; β = 0.23, FDR = 3.23 × 10^−4^ in CSF; β = 0.26, FDR = 4.54 × 10^−4^ in plasma), indicating that higher Cathepsin H levels significantly increase AD risk. Furthermore, our colocalization analysis found the evidence of one functional variant in *CTSH* affecting both Cathepsin H levels and AD risk in all three tissues (posterior probability PP.H4 = 0.995 in brain; PP.H4 = 0.960 in CSF; PP.H4 = 0.948 in plasma).

### Web browser for navigating GWAS and PheWAS results

**Fig. 7:**
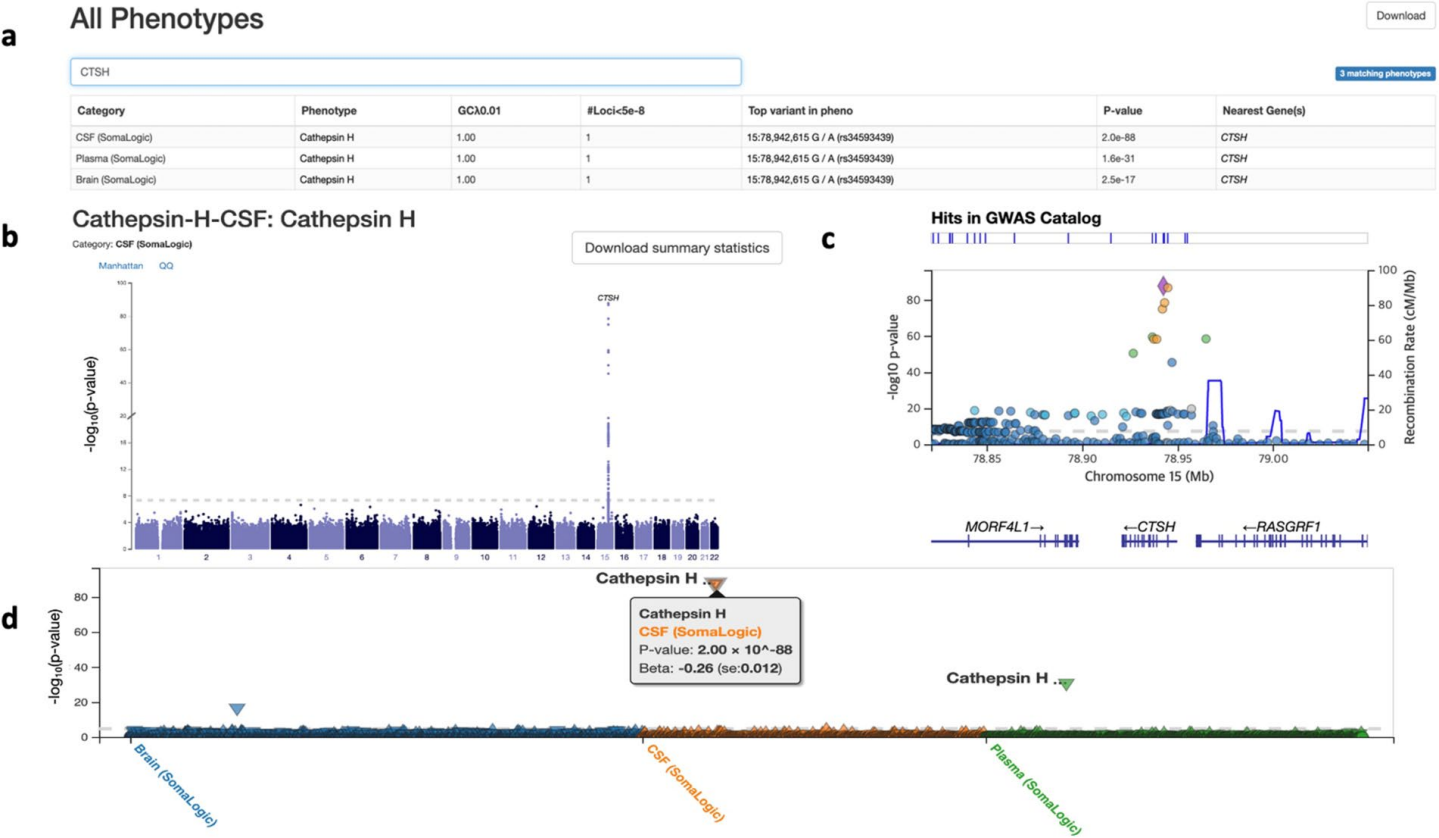
Web browser ONTIME for Cathepsin H protein. The ONTIME browser includes tabular information, where any user can search a particular protein (**a**), a Manhattan plot of Cathepsin H in CSF (**b**), a LocusZoom plot at *CTSH* locus on chromosome 15 (**c**), and a phenome-wide view for the variant rs34593439 showing association with multiple proteins (**d**).

Our pQTL study generated GWAS results for 1300 proteins in brain, 869 proteins in CSF and 953 proteins in plasma, where each GWAS provided an association at about 9 million genetic variants. To enable other interested researchers to navigate the association results from this study, we have now extended our web browser, the Online Neurodegenerative Trait Integrative Multi-Omics Explorer (ONTIME) using PheWeb^28^. The site includes Manhattan plots to display association for each of the 3122 GWAS results and regional view (LocusZoom) plots to visualize association at a particular locus for each protein. In addition, the site provides a phenome-wide association studies (PheWAS) plot for each genetic variant to show association for the variant across all proteins in all three tissues. To illustrate our ONTIME resource, Fig. 7 presents a Manhattan plot of Cathepsin H protein in CSF, a LocusZoom plot at *CTSH* locus on chromosome 15, and a phenome-wide view for the variant rs34593439, showing the consistent associations in all three tissues.

## Discussion

We previously performed pQTL study for protein levels in neurologically relevant tissues and identified tissue-specific pQTLs^6^. We have now expanded and enhanced this work, with an almost two-fold increase in the number of genetic variants (around 9 million variants). In this study, we identified 38 genomic regions associated with 43 proteins in brain, 150 regions associated with 247 proteins in CSF, and 95 regions associated with 145 proteins in plasma. They included *trans*-associated loci for 6 proteins in brain, 52 proteins in CSF, and 47 proteins in plasma. In addition, we have expanded our web portal ONTIME (https://ontime.wustl.edu/) to include this pQTL study for use by the scientific community.

Our comprehensive study uncovering genetic regulation of protein levels provides an opportunity to deliver improved understanding of the mechanistic basis of disease. As a proof of concept, we performed Mendelian randomization and colocalization with the AD GWAS^24^. We identified the evidence for Cathepsin H being causal and colocalized with AD risk at *CTSH* in all three tissues, indicating that higher Cathepsin H levels significantly increase AD risk. Cathepsins, a group of lysosomal proteases, play a central role in several cellular processes including degradation of intracellular proteins, extracellular matrix remodeling, and apoptosis. Cathepsins B, D and E are shown to play a key role in neuroinflammation and ß-Amyloidosis^41–43^. Up-regulated microglial Cathepsin H expression, release, and activity in brain is shown to lead to neuronal death in neuroinflammation^44^. Recently, causality of *CTSH* gene for AD was reported with mass-spectrometry brain proteomic ROS/MAP data^45^. Our findings support this causality in brain and extend it further to CSF and plasma. While we demonstrated this analysis with AD, our pQTL findings are a useful resource for studying neuropsychiatric and neurodegenerative disorders. We hope that this will be valuable for the scientific community.

### Supplementary information

Supplementary Figures

Supplementary Tables

## Data Availability

The genomics data (accession number NG00127.v1) was uploaded to https://dss.niagads.org/datasets/ng00127. The proteomics data and all pQTL results (accession number NG00102.v1) were uploaded to https://www.niagads.org/datasets/ng00102. As these pQTL results are very large, we created multiple Zenodo. The brain pQTL results are stored in four archived files accessible through the following DOIs: 10.5281/zenodo.8190917^29^, 10.5281/zenodo.8190999^30^, 10.5281/zenodo.8191005^31^, and 10.5281/zenodo.8191008^32^. The CSF pQTL results are in three archived files, accessible via the following DOIs: 10.5281/zenodo.8191014^33^, 10.5281/zenodo.8191018^34^, and 10.5281/zenodo.8191027^35^. The plasma pQTL results are available in four archived files with the following DOIs: 10.5281/zenodo.8191032^36^, 10.5281/zenodo.8191048^37^, 10.5281/zenodo.8191052^38^, and 10.5281/zenodo.8191055^39^. In addition, significant pQTL results are provided in a file named ‘pQTL-hg38 Uploaded Tables.xlsx’ (10.5281/zenodo.10011473).

## Code availability

Analysis in this study was performed with the following open-access programs.

plink1.9 v1.90b6.4: https://www.cog-genomics.org/plink/1.9/

plink2 v2.0, alpha software for processing genetic data and performing GWAS: https://www.cog-genomics.org/plink/2.0/

TOPMed genotype imputation on GRC38: https://imputation.biodatacatalyst.nhlbi.nih.gov/

R package liftOver: https://www.bioconductor.org/help/workflows/liftOver/

R package TwoSampleMR: https://mrcieu.github.io/TwoSampleMR/news/index.html

R package Coloc: https://github.com/chr1swallace/coloc

PheWeb v1.1.19, a web server for browsing phenome-wide associations: https://github.com/statgen/pheweb

In addition, we uploaded the R code for conducting Mendelian randomization and colocalization (as the filenames ‘mr.R’ and ‘coloc.R’, resepectiviely) to Zenodo: (10.5281/zenodo.10011473).
