# Supplementary material for: TOPMed imputed genomics enhances genomic atlas of the human proteome in brain, cerebrospinal fluid, and plasma
Source: Sci Data. 2024 Apr 16;11:387. doi: 10.1038/s41597-024-03140-3 (PMC11021418; doi:10.1038/s41597-024-03140-3)

# **TOPMed imputed genomics enhances genomic atlas of the human proteome from brain,** **cerebrospinal fluid, and plasma**

# **Supplementary figures**

## Supplementary Figure 1. PCA plot of Knight-ADRC samples against the 1000 Genomes Project data.

We performed principal component analysis (PCA) after merging the high-quality genomic data of Knight-ADRC participants and the sequencing data from the 1000 Genomes Project (1KG). The scatter plot between first two PCs presents clustering of the Knight-ADRC individuals against samples in the 1KG data. We selected Knight-ADRC samples (shown in gray) that were genetically similar to 522 European individuals from the 1KG data (in magenta). While this plot includes three ancestries from the 1KG data (671 Africans, AFR in blue; 492 South Asians, SAS in cyan; 515 East Asians, EAS, in green), Americans from 1KG were excluded in this figure due to their large admixture.


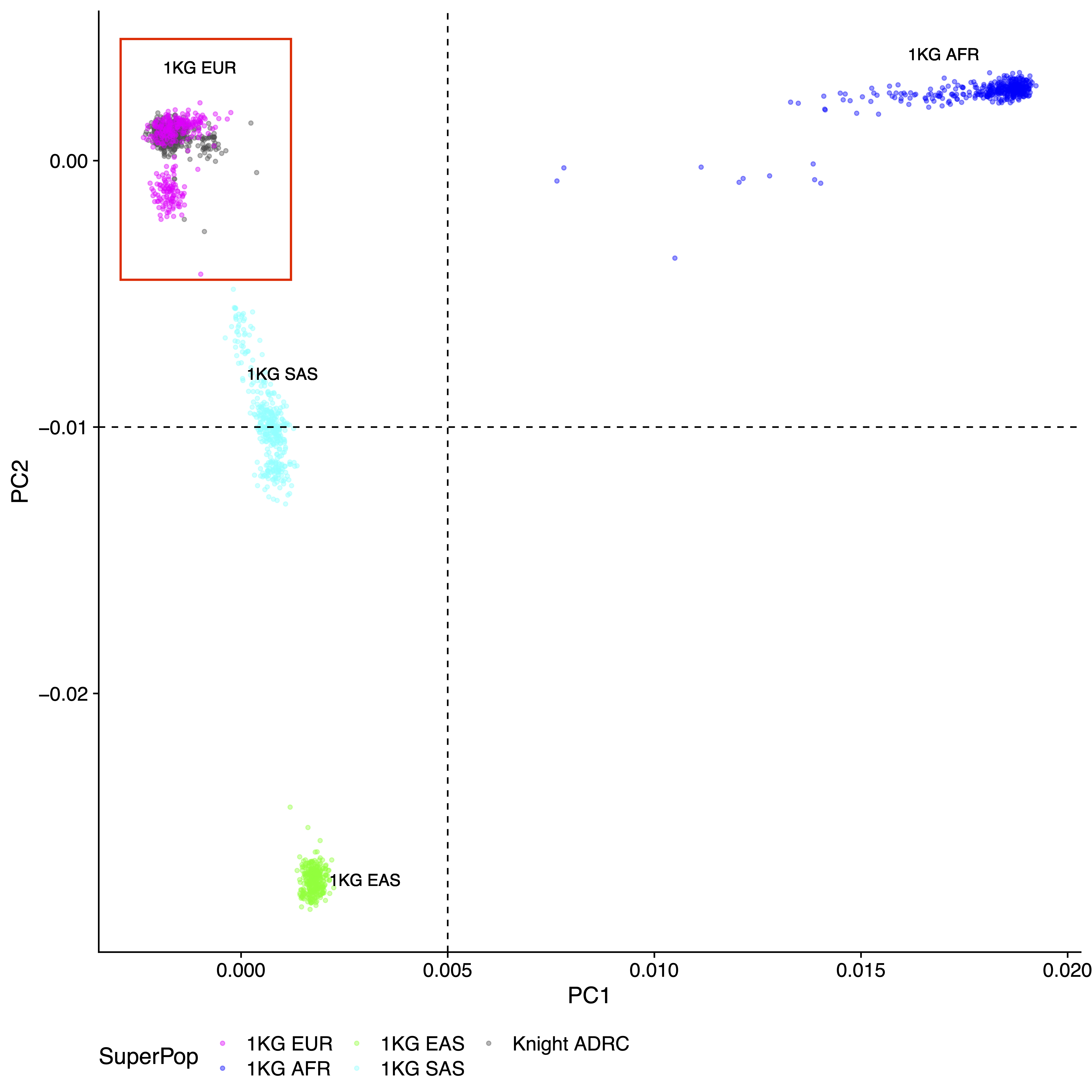


## Supplementary Figure 2. Distribution of brain proteomics data.

Proteins abundance values were log10 transformed for our analysis. These transformed values in brain proteomics data (1300 proteins) are shown here with histogram. Each histogram is labelled by SomaScan Analytes ID. See Supplementary Table 3 for the corresponding protein and gene names.

## Supplementary Figure 3. Distribution of CSF proteomics data.

Proteins abundance values were log10 transformed for our analysis. These transformed values in CSF proteomics data (869 proteins) are shown here with histogram. Each histogram is labelled by SomaScan Analytes ID. See Supplementary Table 3 for the corresponding protein and gene names.


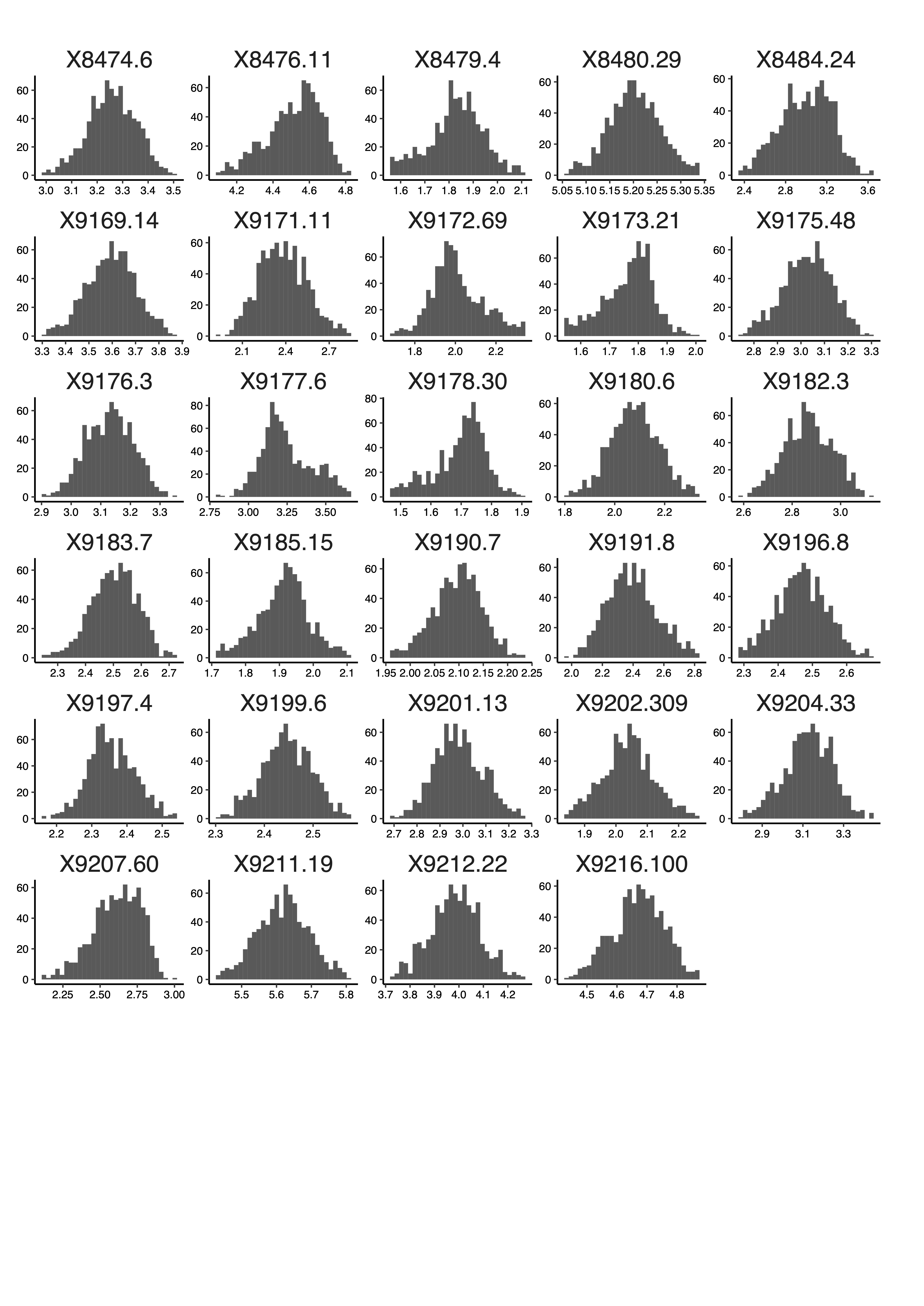

## Supplementary Figure 4. Distribution of plasma proteomics data.

Proteins abundance values were log10 transformed for our analysis. These transformed values in plasma proteomics data (953 proteins) are shown here with histogram. Each histogram is labelled by SomaScan analyte ID. See Supplementary Table 3 for the corresponding protein and gene names.


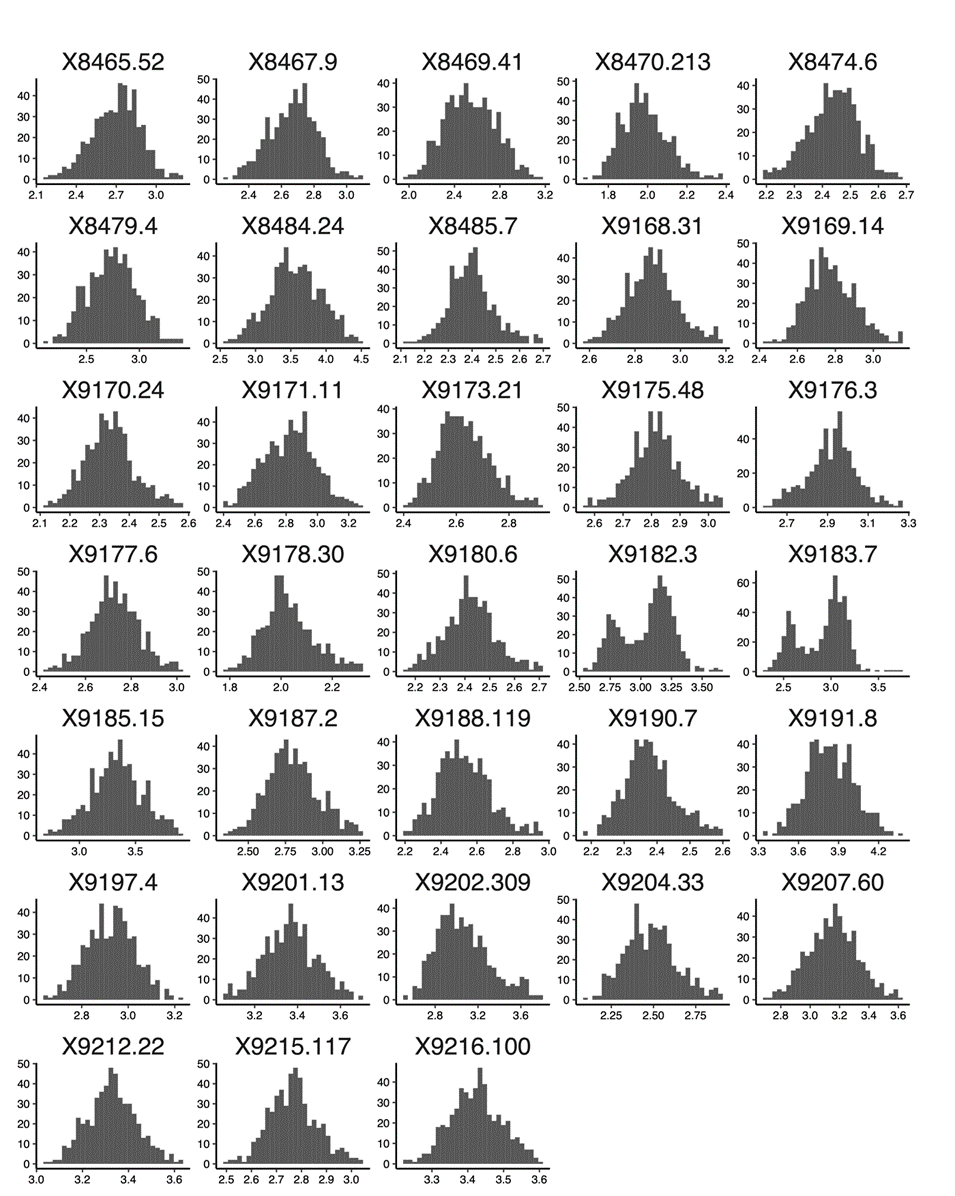

Supplement: Supplementary file 1 — Supplementary Figures [file 41597_2024_3140_MOESM1_ESM.docx]
